# Time to Complete Wound Healing in HIV-Positive and HIV-Negative Men following Medical Male Circumcision in Kisumu, Kenya: A Prospective Cohort Study

**DOI:** 10.1371/journal.pone.0061725

**Published:** 2013-04-15

**Authors:** John H. Rogers, Elijah Odoyo-June, Walter Jaoko, Robert C. Bailey

**Affiliations:** 1 University of Illinois at Chicago, Chicago, Illinois, United States of America; 2 Nyanza Reproductive Health Society, Kisumu, Kenya; 3 University of Nairobi, Nairobi, Kenya; University of Buea, Cameroon

## Abstract

**Background:**

While voluntary medical male circumcision (VMMC) has been shown to be protective against HIV-acquisition, the procedure may place men and their partners at risk of HIV infection in the period following circumcision if sex is resumed before the wound is healed. This prospective cohort study evaluates post-circumcision wound healing to determine whether the 42-day post-circumcision abstinence period, recommended by the World Health Organization and adopted by VMMC programs, is optimal.

**Methods and Findings:**

Men were circumcised by forceps-guided method and their post-circumcision wounds examined weekly for seven weeks and at 12 weeks. Time to complete healing was recorded in completed weeks since circumcision, and its associations with baseline covariates were assessed by Kaplan-Meier methods and Cox Proportional Hazard Models. A total of 215 HIV-negative and 108 HIV-positive men aged 18–35 years (median 26, IQR 23–30) were enrolled. 97.1% of scheduled follow-up visits were completed. At week 4, 59.3% of HIV-positive men and 70.4% of age-matched HIV-negative men were healed. At week 6, these percentages rose to 93.4% in HIV-positive men and 92.6% in age-matched HIV-negative men. There was no difference in the hazard of healing between 108 HIV-positive and 108 age-matched HIV-negative men (HR 0.91 95% CI 0.70–1.20). Early post-operative infection was associated with delayed healing in both HIV-positive and HIV-negative men (HR 0.48 95% CI 0.23–1.00).

**Conclusions:**

Our results indicate that the WHO recommendation for 42-days post-circumcision sexual abstinence should be maintained for both HIV-positive and HIV-negative men. It is important to stress condom use upon resumption of sex in all men undergoing circumcision.

## Introduction

Three randomized controlled trials in Kisumu, Kenya, Rakai, Uganda, and Orange Farm, South Africa showed that medical male circumcision reduces the risk of HIV-acquisition by 60%. [1], [2], [3] Male circumcision is now a recommended component of a comprehensive prevention package for men in communities with high HIV-prevalence, low circumcision rates, and where heterosexual sex is the primary mode of transmission. [4] Circumcision involves the surgical excision of the prepuce which results in a circumferential, full-thickness cutaneous, incisional wound near the junction of the glans and the shaft of the penis. Wound healing is initiated via primary intention, wherein the edges of the wound are pulled together, closed, and sutured at the time of surgery. While voluntary medical male circumcision (VMMC) has been shown to be protective in preventing HIV-acquisition, there is concern that the procedure may place men and their partners at higher risk of HIV-acquisition in the period immediately following the circumcision before the wound is healed. [5] During the inflammatory and early proliferative stages of healing, the wound is open to the environment and the integrity of the dermis is compromised. If sexual activity is initiated during these early phases, HIV-negative men may be more susceptible to acquiring HIV as the wound provides an easy portal of entry in an area with a potentially high-concentration of HIV-target cells. [6], [7] Similarly, HIV-positive men with unhealed wounds may have high viral shedding, placing their sex partners at high risk of infection. To allow for complete healing, the World Health Organization recommends 42-days of post-circumcision abstinence. [4].

To date, no published study has systematically investigated the length of time required for the wound to fully heal following circumcision. The Kisumu trial reported complete healing in 98.7% of men returning for their 30-day follow-up visit, [1] and Rakai reported 88.4% of 18–24 year old men and 86.1% of 15–49 year old individuals with complete healing at four weeks post surgery. [5] Neither of these trials used a stringent follow-up protocol for assessment of wounds; the timing of observations of wounds classified as either 30-days or 4-weeks after surgery varied widely, making true assessment of proportions healed at specific time points difficult. There is concern that the 42-day post-circumcision abstinence period may discourage men, particularly those in committed relationships, from seeking circumcision services. Studies of Kenyan men having undergone VMMC have revealed that between 30.9% and 37.7% of men resumed sex before the recommended six weeks abstinence period had elapsed. [8], [9] Higher proportions of married than unmarried men have been shown to resume sex early, prior to the completion of the recommended post-surgery abstinence period. [5], [9], [10] Men’s resumption of sex earlier than recommended may suggest that the imposed abstinence period could be a barrier to uptake of circumcision services, especially among married men or those with a steady sexual partner. Conversely, because we do not yet know the true proportion of men healed by 42 days post-surgery, the recommended abstinence period may be insufficient to protect men and their partners from increased exposure to HIV. In a study of HIV-transmission among serodiscordant couples, Wawer et al reported nearly a three-fold increase in HIV-incidence among female partners of men who resumed sex before certification of healing (RR 2.92 95% CI 1.01–8.46). [11] Additionally, Kigozi et al report a significant difference in healing rates between HIV-positive (92.7%) and HIV-negative (95.8%) men at 6-weeks following surgery (p<0.007). [12] Because of the potential risk to seronegative partners, the possibility that HIV-positive men heal slower than HIV-negative men, and that UNAIDS recommends HIV-positive men be provided VMMC on request if there are no contraindications, we have included HIV-positive men in this study.

This prospective cohort study was undertaken to evaluate post-circumcision wound healing in HIV-positive and HIV-negative men. We examined demographic, behavioral, biological, and surgical factors that influence healing progression with the goal of determining whether the recommended 42-day post-circumcision abstinence period, currently recommended by the World Health Organization and employed by the Kenyan Ministry of Health, is optimal. [4].

## Methods

### Participants

The study was conducted at the Universities of Nairobi, Illinois, and Manitoba (UNIM) Clinic within Lumumba Health Center in Kisumu, Kenya. Participants were selected from men seeking voluntary medical male circumcision (VMMC). These men received a study flier that expressed the goals of the Wound Healing Study (WHS) and were told that their eligibility for VMMC would not be affected if they chose not to participate in the WHS. Men expressing interest in the WHS then underwent voluntary HIV testing and counseling (VCT) and were screened for circumcision status.

Men aged 18–35 years were eligible for enrollment if they were uncircumcised, had no contraindications for circumcision, were a resident of Kisumu, and intended to stay in Kisumu for a period of 3-months following VMMC. Consistent with Kenyan VMMC guidelines, HIV-positive men with signs of advanced HIV-infection (WHO Stage 3 or greater) were excluded. Because age may be associated with time to healing and HIV-status, we matched HIV-positive individuals to an HIV-negative individual of the same age +/−2 years. Because most men in VMMC programs are HIV-negative, additional HIV-negative men were enrolled to increase precision in this group. Analysis of all the HIV-negative men was performed separately. This study was approved by the Institutional Review Board at the University of Illinois at Chicago, USA and the Ethics and Research Committee at Kenyatta National Hospital, Kenya. All participants provided written informed consent.

### Procedures

After giving informed consent, enrolled men underwent the same pre-operative assessment that is in place for Kenya MOH circumcision programs. Additionally, men were tested for HSV-2 serostatus, random blood sugar level, hemoglobin level, serum albumin level, and were given an extensive interview on behavioral and demographic characteristics. HIV-positive men also had blood drawn for CD4+ T cell count. Men who completed the enrollment interview and passed the pre-operative assessment, which included a physical examination, were then circumcised by one of four clinicians (2 clinical officers and 2 nurses). In order to control for surgeon experience, a possibly influential covariate with time to healing [10], each of the study surgeons was hired because he had previously performed several hundred circumcisions either under the Kenya MOH program or as part of previous studies at the UNIM clinic. All surgeries were performed using the forceps-guided method for male circumcision. [4].

Following circumcision, men were instructed to return on a specific date every 7-days from the date of operation for seven weeks and then a final visit at 12 weeks. At each follow-up visit the wound was assessed for progression through the healing process, photographs were taken to document healing and adverse events, and the participant was interviewed to collect data on post-operative behavior and satisfaction with the healing process. Visits were eligible to be included in analysis if they occurred on the scheduled day +/−2 days. If a participant missed his scheduled follow-up visit, a trace was implemented to locate the individual and ensure his attendance the following day. In this way, we were able to complete 97.1% (2510/2584) of scheduled visits. The primary outcome of complete wound healing was assessed by clinicians using the following operational definition: healthy scar formation with no scab or opening along the incision line. Prior to beginning the study and to ensure adherence to the definition of a completely healed wound, our clinicians underwent extensive training and pilot testing where individuals with wounds of varying post-operative age were assessed by each clinician and discussed as a group.

### Statistical Analysis

A participant could not be certified as healed until he was examined by one of the study clinicians. The number of post-operative days elapsed at the time of certified complete healing was recorded for each individual. Clinic visits proceeded after certified complete healing and as such it was possible for a participant to be certified as completely healed at one visit and not be certified as completely healed at the next visit. This scenario occurred in 11 of 323 individuals. For analysis purposes, an individual could not be considered completely healed until he was certified healed at all subsequent visits. Censored individuals were those that were either lost-to-follow-up before certified healing or that completed the full 12 weeks of follow-up without accomplishing complete healing, which occurred in just one individual.

We performed two separate series of analyses. First, we examined the relationship between HIV-status and time to complete healing in an age-matched cohort of 108 HIV-positive and 108 HIV-negative individuals. Second, we examined time to healing in 215 HIV-negative men. Our sample size of 108 pairs was sufficient to detect a 12% or greater difference in the proportion of completely healed men between the two groups at alpha = 0.05 and power = 80%. Kaplan-Meier methods were used to assess time to complete wound healing in the matched cohort stratified by HIV-status. Cox proportional hazard models were used in both series to examine the relationship between *a priori* identified potentially important covariates and the hazard of healing at any time point. These covariates fall into four categories: demographic, biological, behavioral, and surgical. Demographic characteristics considered were age and marital status. Biological characteristics were: HIV-status, HSV2 status, baseline random blood sugar, hemoglobin, serum albumin, and the presence of a post-operative infection in the first 3 weeks of follow-up. Behavioral characteristics were: baseline alcohol consumption (days/week), physical activity in the first week following surgery (riding a bicycle, digging, and walking long distances), and time of onset of sexual activity. Surgical characteristics were: amount of dermis exposed at week 1 (in total mm), evidence of tight sutures at week 1, surgical time as a proxy for difficult surgery, and surgeon cadre (clinical officer vs. nurse counselor).

We investigated each of the covariates individually in separate univariate models to explore the reduction in −2 log likelihoods using a one-degree of freedom chi-square test. Covariates significantly reducing the model deviance at the p<0.05 level were then included in a multivariate Cox Proportional Hazard model. Covariates failing to meet this criterion were dropped from further analysis with the exception of the primary exposures of interest: HIV-status for matched analysis and age for HIV-negative analysis. We applied this strategy with both the age-matched analysis of HIV-positive and HIV-negative men and with the analysis of HIV-negative men only. In each analysis, we modeled the hazard of healing and thus hazard ratios less than 1.0 are indicative of delayed healing. Analyses were performed using SAS version 9.2 (SAS Institute, Cary, NC).

## Results

### Participant Characteristics

A total of 215 HIV-negative and 108 HIV-positive men aged 18–35 years (median 26 years, IQR 23–30) were enrolled. Nearly half of all men (46%) were married; 58% of HIV-positive men were married. HSV-2 serology was positive in 75% of HIV-positive men and 36% of HIV-negative men. Almost all men (95%) had random blood sugar levels in the normal range (70–139 mg/dL); 91% of men had normal hemoglobin levels (≥13.0 g/dl); and 94% of all men had serum albumin levels in the normal range (3.40–5.40 g/dL). Among HIV-positive men, 46/108 (43%) had CD4 counts below 350 cells/µL. Each of the 323 participants was scheduled for weekly post-circumcision visits till week 7 and at week 12 giving a total of 2,584 expected visits, of which 2,510 (97.1%) were completed. Baseline characteristics are provided in Table 1.

**Table 1:** Characteristics of HIV-positive (HIV+) men, their HIV-negative (HIV-) matches, and all HIV-negative men at enrollment.

		HIV+	HIV−	p-value	All HIV-
Characteristic	Categories	(n = 108)	(n = 108)	(matched)	(n = 215)
Enrollment age (years)	18–24	22 (20.4%)	22 (20.4%)	–	89 (41.4%)
	25+	86 (79.6%)	86 (79.6%)		126 (58.6%)
Marital status	Single	45 (41.7%)	50 (46.3%)	p = 0.49	128 (59.5%)
	Married	63 (58.3%)	58 (53.7%)		87 (40.5%)
Sexual partners in past year	None	5 (4.7%)	0 (0.0%)	p = 0.06	4 (1.9%)
	One	42 (39.3%)	45 (42.4%)		96 (45.3%)
	Two	27 (25.2%)	36 (34.0%)		64 (30.2%)
	Three or more	33 (30.8%)	25 (23.6%)		48 (22.6%)
Alcohol use (days per week)	None	63 (58.3%)	51 (47.2%)	p = 0.18	116 (53.9%)
	Less than one	11 (10.2%)	8 (7.4%)		21 (9.8%)
	One to two	20 (18.5%)	25 (23.2%)		45 (20.9%)
	Three or more	14 (13.0%)	24 (22.2%)		33 (15.4%)
HSV-2 serology	Positive	81 (75.0%)	50 (46.3%)	p<0.001	78 (36.3%)
	Negative	27 (25.0%	58 (53.7%)		137 (63.7%)
Random blood sugar (mg/dL)	<70	0 (0.0%)	3 (2.8%)	p = 0.22	4 (1.9%)
	70–139	103 (95.4%)	102 (94.4%)		203 (94.4%)
	140–200	4 (3.7%)	2 (1.9%)		6 (2.8%)
	>200	1 (0.9%)	1 (0.9%)		2 (0.9%)
Hemoglobin (g/dL)	<13.0	20 (18.5%)	4 (3.7%)	p<0.001	10 (4.7%)
	≥13.0	88 (82.5%)	104 (96.3%)		205 (95.3%)
Serum albumin (g/dL)	<3.40	10 (9.3%)	1 (0.9%)	p<0.01	5 (2.3%)
	3.40–5.40	97 (89.8%)	105 (97.2%)		208 (96.7%)
	>5.40	1 (0.9%)	2 (1.9%)		2 (0.9%)
CD4 count at baseline (cells/µL)	>350	61 (57.0%)	–	–	–
	≤350	46 (43.0%)			
ART at baseline	Yes	41 (38.0%)	–	–	–
	No	67 (62.0%)	–	–	–

p-values are for the difference between HIV+ and HIV− matches.

### Time to Complete Healing

**Figure 1:**
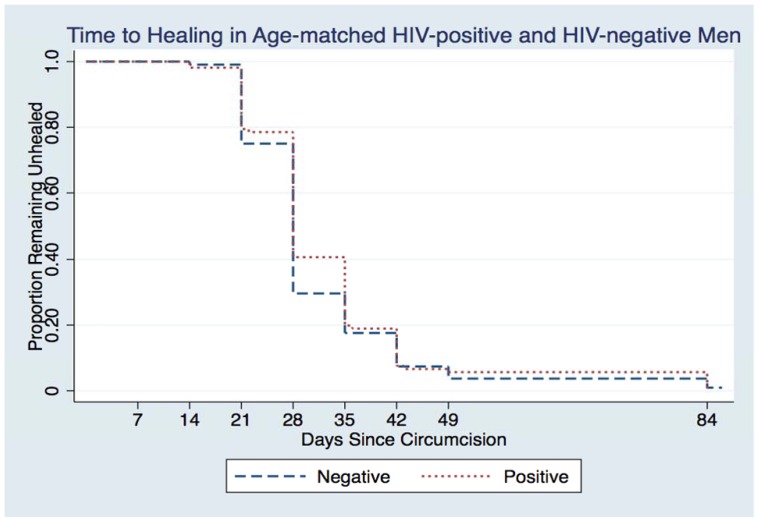
Time to complete wound healing in age-matched HIV-positive and HIV-negative individuals (log-rank = 0.69, p = 0.41).

Figure 1 provides the results of the Kaplan-Meier analysis for the age-matched cohort. No statistically significant difference in time to complete healing by HIV-status was observed (log-rank test = 0.69, p = 0.41). Mean time to complete healing was 33 days for HIV-positive individuals, 31 days for HIV-negative matches, and 31 days for all HIV-negative individuals. One HIV-negative individual failed to accomplish complete healing by the end of the follow-up period at 12 weeks due to an adverse event (sub-dermal hematoma) in the first post-operative week. This was the only severe adverse event observed in the 323 individuals (0.3%).

By the week 4 visit, 64.7% of all men were completely healed. This figure rose to 83.1% at week 5, 94.1% at week 6, and 96.6% at week 7 (Table 2). The largest disparity in complete healing between HIV-negative and HIV-positive men occurred at week 4 when 70.4% of HIV-negative and 59.3% of HIV-positive men were healed (p = 0.09). By week 6 cumulative percentage of healed individuals reached 93.3% in HIV-positive men, 92.6% in age-matched HIV-negative men, and 94.4% in all HIV-negative individuals. Mean time to complete healing was 34.5 days in HIV-positive men with baseline CD4 counts less than or equal to 350 cells/µL and 31.9 days in men with baseline CD4 counts greater than 350 cells/µL (p = 0.20). Median time to complete healing was 28 days for both groups. Among ARV-naïve HIV-positive men, 59.7% (40/67) had baseline CD4 counts greater than 350 cells/µL. Mean time to complete healing was 31.1 days in this group versus 37.1 days in ARV-naïve men with baseline CD4 counts less than or equal to 350 cells/µL (p = 0.04). There was no difference at week 6 between those with CD4 counts above versus those below 350 cells/µL (94.7% vs 88.9%; p = 0.64).

**Table 2:** The cumulative proportion of HIV-positive and HIV-negative men healed at weekly intervals following medical male circumcision.

	HIV-positive (n = 108)		HIV-negative matches (n = 108)		All HIV-negative (n = 215)	
Follow-up week	n returning for visit(%)	Cumulative% healed	n returning for visit (%)	Cumulative% healed	n returning for visit (%)	Cumulative % healed
Week 1	108 (100)	0.0	107 (99.1)	0.0	213 (99.1)	0.0
Week 2	104 (96.3)	1.9	106 (98.1)	0.9	212 (98.6)	1.4
Week 3	105 (97.2)	21.5	107 (99.1)	25.0	212 (98.6)	23.8
Week 4	103 (95.3)	59.3	108 (100)	70.4	212 (98.6)	67.3
Week 5	104 (96.3)	81.1	107 (99.1)	82.4	212 (98.6)	84.1
Week 6	101 (93.5)	93.4	107 (99.1)	92.6	212 (98.6)	94.4
Week 7	100 (92.6)	94.3	105 (97.2)	96.3	210 (97.7)	97.7
Week 12	97 (89.8)	100.0	103 (95.3)	99.1	205 (94.0)	99.5

### HIV-positive Versus Age-matched HIV-negative Men

In the age-matched cohort of HIV-positive and HIV-negative men, mean time to complete healing was 45.9 days in individuals presenting with an early post-operative infection versus 30.9 days in men who did not (p<0.001). Under univariate analysis early post-operative infection resulted in a significantly reduced rate of complete healing (HR 0.53, 95% CI 0.32–0.89) (Table 3). At six-weeks post-circumcision, 72.2% of men with early post-operative infections were healed versus 94.9% of men without infections (p<0.001). Men presenting with evidence of tight sutures at week 1 also had reduced rates of healing (HR 0.65 95% CI 0.42–0.996), but at six-weeks post-circumcision the difference in proportions healed between men without and with evidence of tight sutures was non-significant (94.2% vs. 83.3% p = 0.07). This variable, along with early post-operative infection, was included in multivariate modeling. HIV-status, although not significantly associated with time to healing, was also included in the multivariate model. In the multivariate Cox proportional hazard model, early post-operative infection was associated with a reduction in the rate of healing (HR 0.52 95% CI 0.31–0.87) and men with evidence of tight sutures also experienced reduced rates of healing (HR 0.63 95% CI 0.41–0.98).

**Table 3:** Results of univariate Cox Proportional Hazard Models of the hazard of complete healing by demographic, biological, behavioral and surgical variables.

		Age-matched analysis	All HIV-negative men only
Category	Covariate	HR (95% C.I.)	HR (95% C.I.)
Demographic	Age 25+	–	0.91 (0.69–1.19)*
	Married	0.94 (0.71–1.23)	0.86 (0.66–1.14)
Biological	HIV-Positive	0.92 (0.70–1.20)*	–
	HSV2-Positive	0.99 (0.75–1.31)	1.06 (0.80–1.41)
	Blood sugar ≥140 mg/dl	0.77 (0.38–1.55)	0.63 (0.31–1.28)
	Hemoglobin ≥13.0 g/dl	0.95 (0.62–1.45)	0.70 (0.37–1.32)
	Serum albumin ≤3.39 g/dl	0.92 (0.50–1.69)	0.95 (0.39–2.31)
	Post-operative infection	0.53 (0.32–0.89)*	0.48 (0.23–1.00)*
Behavioral	Alcohol consumption ≥3 days/week	0.79 (0.56–1.13)	0.87 (0.60–1.26)
	Physical activity at week 1	0.94 (0.64–1.40)	1.15 (0.76–1.74)
	Sexual activity before week 3	0.92 (0.53–1.62)	1.10 (0.58–2.08)
Surgical	Dermis exposed at week 1≥5.0 mm	0.60 (0.33–1.12)	0.86 (0.35–2.12)
	Tight sutures at week 1	0.65 (0.42–1.00)*	0.80 (0.53–1.22)
	Surgical time (continuous)	1.02 (1.00–1.03)	1.01 (0.99–1.03)
	Nurse vs. clinical officer	1.01 (0.73–1.40)	1.20 (0.88–1.64)

* Indicates variables included in multivariate models due either to being a primary exposure of interest or a covariate significantly associated with time to healing at the p<0.05 level.

### HIV-negative Men

Exploration of age in HIV-negative men as an explanatory factor for either accelerated or reduced rates of healing yielded non-significant results (HR 0.91 95% CI 0.69–1.19). As with the age-matched analysis, early post-operative infection was associated with reduced rates of healing (HR 0.48 95% CI 0.23–1.00). Mean time to healing was 30.2 days in men with no infection and 46.7 days in men presenting with an infection (p = 0.005). At six-weeks post-circumcision, 95.6% of men without an infection were certified healed versus 66.7% of those with early post-operative infections (p = 0.009). In multivariate Cox proportional hazard models early post-operative infections maintained a significant reduction in healing rates while adjusting for age (HR 0.48 95% CI 0.23–1.00). Unlike the age-matched analysis, tight suturing was not associated with delays in healing (HR 0.80 95% CI 0.53–1.22).

## Discussion

We found that 94% of men are completely healed within the 42 day time period recommended by WHO for post-surgical abstinence. Our finding of 64.7% healed at week 4 is lower than the proportion healed at 30-days in the Kisumu trial (98.7%) and week 4 in the Rakai trial (86.1%). Neither of these trials used a stringent follow-up protocol for assessment of wounds; the timing of observations classified as 30-days or 4-weeks post-surgery varied widely. In the Kisumu trial, 5.3% of wounds were assessed at or before 27 post-operative days, 89.7% were assessed at between 28 and 34 days, and the remaining 5.0% were assessed after at least 35 days. [1] Similarly, in the Rakai trial, among all participants, 25.5% of men were seen at or before 27 days, 69.5% at 28–34 days, and 5.1% after 35 days. [5] In the Kisumu trial, suturing was performed using 4.0 sutures, which may provide better apposition of the wound edges compared with the 3.0 sutures used in this study and in the Kenyan national VMMC program. We also used a slightly different definition of complete wound healing from the trials. In the Kisumu trial, a completely healed wound was defined as a wound without a scab or open wound and no evidence of swelling or redness. [5] The Rakai definition emphasized the formation of an intact, healthy scar with no residual exudate or scab formation and the complete absorption of all sutures. [2] Our definition, healthy scar formation with no scab or opening along the incision line, was formulated by the clinical team. Color was eliminated from the definition as we found wide variability in the color of healthy, fully healed tissue during pilot testing. Having all the sutures absorbed was unnecessary language, since this occurs early in the post-circumcision period. Therefore, differences between our findings and those reported for trial participants may be a function of different definitions of complete healing, the requirement in our study that men were seen in seven-day intervals (+/−2 days), and the requirement that a man be considered healed only after being recorded as healed at all subsequent follow-up visits. Kigozi et al report that 92.7% of HIV-positive men and 95.8% of HIV-negative men were healed at 6-weeks following surgery (p<0.007), [12] results that closely approximate our findings, although our rates of 93.3% in HIV-positive and 94.4% in HIV-negative men were not significantly different.

In assessing whether a change in the WHO guidelines is recommended, the results from this wound healing study suggest that the 42-days of recommended post-circumcision abstinence is reasonable and should be maintained. While most men are healed at week 4 and week 5, there is significant healing that takes place between 28 and 42 days; nearly thirty-percent of men healed in this period (29.6%). Additionally, since 94% of men are healed by 42-days, extending the abstinence recommendation to 7 weeks or beyond may present an additional barrier to uptake of circumcision with limited returns in the form of reduced HIV-incidence. Rather than extend the abstinence period, it would be prudent to reinforce counseling for clients and to stress consistent condom use when sex is resumed.

Our results are specific to the forceps-guided method of surgical circumcision. Recent attention to the use of devises for circumcision may require that we consider the WHO guidelines for post-circumcision abstinence in the context of these devices. A recent randomized controlled trial of the Shang Ring versus surgical circumcision in Kenya and Zambia reported 76% of men undergoing the Shang Ring procedure were healed at 42-days post-circumcision, compared to 85% of men undergoing surgical circumcision. [13] With nearly a quarter of the Shang Ring population unhealed at 42-days, a revision to the WHO recommended post-circumcision abstinence period may be necessary. In a study of the Prepex device for male circumcision in Rwanda, the authors report that “the number of days required for complete healing” following device removal was 31 days, or approximately 38 days from device placement. [14] Assuming that the authors are reporting median time to healing, circumcision with Prepex may require a longer healing period than either the forceps-guided method or the Shang Ring. This may be expected as circumcision with the Prepex device requires healing by secondary intention. Unfortunately, the authors fail to report healing rates at 42-days post-circumcision.

We found that HIV-status was not associated with a significantly longer healing period in this population. However, within ARV-naïve HIV-positive men, those with CD4 counts of less than 350 cells/µL experienced slower healing than those with counts above 350 cells/µL. These findings suggest that men of known HIV-positive status who are not on ARVs should be counseled to refrain from sex for longer than the 42 day recommended period and, as with all men, counseled to use a condom with resumption of sex.

All other patient-centered covariates were not significantly related to time to healing. Variables associated with surgery proved most influential; namely, evidence of tight suturing and post-operative infection, the later being related perhaps to poor hygiene and post-operative wound care or contamination at the time of surgery. In analysis of HIV-negative men the variable most associated with hazard of delayed healing was post-operative infection.

We failed to account for hygiene covariates during the course of the study. Carriage of the penis following surgery, where the dorsal surface is held against the lower abdomen to avoid postural edema around the wound, and post-surgical washing may influence the wound’s ability to heal. Investigating these covariates will be helpful for further understanding of post-circumcision healing. We introduced selection bias to the study by age-matching HIV-negative men to enrolled HIV-positive men. HIV-status is linked to age in this population. As a result, we have an HIV-negative study population that is older than what is reflected in men seeking circumcision under the Kenya National VMMC Program. Although our clinicians are instructed to follow a strict protocol with regards to assessment of each wound, there exists the possibility that the number of completed weeks of follow-up influenced the clinicians’ assessment of each wound. We have accounted for this possibility by requiring that each wound be certified healed at all subsequent study visits. Additionally, because the clinicians conducting the wound assessments were the same as those performing the surgery, it is possible that they may be biased towards reporting earlier healing. However, each participant could see any one of the four clinicians at each visit. Also, it was not feasible to employ additional clinicians for independent follow-up assessments. We required our clinicians to attend an extensive training program and adherence to the definition of complete wound healing was piloted repeatedly before onset of the study.

Infection in the first three weeks post-circumcision and evidence of tight suturing at the first follow-up visit were associated with delayed healing. When these events are observed after surgery, clients should be counseled that they may need to remain abstinent beyond 42-days and that they should use condoms upon resumption of sex. Additionally, HIV-positive men who are ARV-naïve might be counseled to refrain from sex longer than 42-days. As with counseling for all men undergoing circumcision, emphasis should be placed on condom use at resumption of sex.

This is the first study to assess systematically wound progression following voluntary medical male circumcision by forceps-guided method. Based on our results, the WHO recommendation for 42-days post-circumcision abstinence should be maintained and is appropriate for HIV-positive and HIV-negative men since we found no difference in time to complete wound healing between these groups. We have identified factors associated with delayed wound healing - post-operative infection, tight sutures, and low CD4 counts in ARV-naive HIV-positive patients - that should be taken into account in training for and implementing VMMC programs.
